# Barriers and Facilitators to HIV and Viral Hepatitis Testing in Primary Health Care Settings in the Kyrgyz Republic (BarTest): Protocol for a Mixed Methods Study

**DOI:** 10.2196/62929

**Published:** 2025-05-28

**Authors:** Ida Sperle, Nikolay Lunchenkov, Zuridin S Nurmatov, Aybek A Bekbolotov, Anastassiya Stepanovich-Falke, Michael Brandl, Olena Kysil, Stela Bivol, Viviane Bremer, Barbara Gunsenheimer-Bartmeyer, Sandra Dudareva

**Affiliations:** 1 Department of Infectious Disease Epidemiology Robert Koch Institute Berlin Germany; 2 TUM School of Social Science and Technology Technical University of Munich Munich Germany; 3 National Institute of Public Health of the Ministry of Health Bishkek Kyrgyzstan; 4 World Health Organization, Regional Office for Europe Copenhagen Denmark; 5 Institute of Public Health Riga Stradiņš University Riga Latvia

**Keywords:** HIV, viral hepatitis, testing, barriers, health care workers, Kyrgyz Republic, primary health care

## Abstract

**Background:**

In the Kyrgyz Republic, it is estimated that 18% of people living with HIV are undiagnosed and more than half are diagnosed late (CD4 lymphocyte count of <350 cells/μL). For viral hepatitis, before 2023, free testing was only available to people living with HIV, which led to a low testing uptake. A new national program on the elimination of HIV and viral hepatitis infection for the years 2023-2027 recognizes the need to scale up testing to reduce the gap in undiagnosed people in the country.

**Objective:**

This study aimed to identify and describe the most important barriers and facilitators to HIV and viral hepatitis B, C, and D testing from the perspective of health care workers working in primary health care settings in the Kyrgyz Republic.

**Methods:**

A cross-sectional, mixed methods study was conducted in 2 phases. A purposive sampling approach was applied to recruit health care workers in primary health care settings. In phase I, in-depth, semistructured interviews were conducted with 22 participants to gather detailed information about the key barriers and facilitators for testing. We applied a thematic approach for qualitative analysis. The themes identified informed the development of a questionnaire with the main barriers and facilitators for phase II. The questionnaire was distributed electronically, and the target sample size was 400 participants. We performed descriptive analyses of the questionnaire data, reporting the most frequently mentioned barriers and facilitators for HIV and viral hepatitis testing.

**Results:**

The study received financial support in the framework of the Global Health Protection Programme by the Federal Government of Germany. Data collection took place in June 2024 for phase I and in November 2024 for phase II. Data analyses and writing up of results will be done in early 2025 and results are expected to be published in spring 2025.

**Conclusions:**

The results of the study will improve the understanding of existing barriers and facilitators to HIV and viral hepatitis testing in order to increase testing offers and uptake in primary health care settings in the Kyrgyz Republic. Importantly, the findings will inform steps to improve the implementation of the new testing strategy and, ultimately, increase the number of people diagnosed and treated in the Kyrgyz Republic.

**International Registered Report Identifier (IRRID):**

PRR1-10.2196/62929

## Introduction

HIV and viral hepatitis remain large public health challenges in the World Health Organization (WHO) European region. An estimated 3 million people are living with HIV, 10.6 million with hepatitis B, and 8.6 million people with hepatitis C [1-3]. In the European region, the public health burden of HIV and hepatitis B and C is particularly high in the Eastern European and Central Asian countries [4].

HIV and viral hepatitis share similar modes of transmission and coinfections are common [5]. For both diseases, a large proportion of those living with the diseases remain undiagnosed due to the often asymptomatic nature of the infections [5,6]. For HIV, around 77% are aware of their HIV status in the WHO European region [7]. For viral hepatitis, an estimated 16% and 29% were diagnosed with hepatitis B and hepatitis C by the end of 2022, respectively [3,5]. There is a large need to scale up testing offers and uptake to reduce the number of people who are undiagnosed and people who are diagnosed late (CD4 lymphocyte count <350 cells/μL), which accounts for 50% in the WHO European Region [8]). This is important not only to facilitate linkage to care and improve individual health but also public health by reducing onward transmission.

WHO has published regional action plans for ending AIDS, viral hepatitis, and sexually transmitted infections for the period 2022-2030 [9] to reach elimination goals by 2030. While some progress has been made, challenges and gaps in reaching elimination remain. One strategic direction in the WHO strategy emphasizes a people-centered approach, and importantly integration and decentralization of services for HIV and viral hepatitis (and other sexually transmitted diseases) in primary health care (PHC) settings where appropriate. A people-centered approach and offering testing for more than one disease has the potential to increase testing uptake, for example, by implementing rapid point-of-care multiplex tests for HIV and viral hepatitis. The strategy also defines testing targets needed to reach elimination by 2030. For HIV, 95% of those living with HIV should know their status and 90% of those living with hepatitis B or hepatitis C should be diagnosed [9].

In the Kyrgyz Republic, it is estimated that 18% of people living with HIV are undiagnosed [10] and more than half are diagnosed late. For viral hepatitis, before 2023, free testing was only available to people living with HIV, which has led to a low testing uptake, further worsened during the COVID-19 pandemic [10]. During a WHO HIV and viral hepatitis program review in the Kyrgyz Republic in April 2022 [10], one priority recommendation was to make use of the experience and structure of the HIV program to optimize synergies, human resources, and the infrastructure for both HIV and viral hepatitis and move away from vertical program structures. It was also recommended to scale up screening for HIV in health care settings by testing patients presenting with conditions that are either AIDS-defining or associated with an undiagnosed HIV prevalence of >0.1% (HIV indicator conditions) [10-12]. HIV and viral hepatitis are political priorities in the Kyrgyz Republic, and a program of the Cabinet of Ministers on the elimination of HIV and viral hepatitis infection for the years 2023-2027 has been adopted [10]. The program recognizes the need to scale up testing and that stigma and discrimination are large barriers, in particular for key populations, for coming forward for testing. The program outlines several steps to improve testing uptake in the Kyrgyz Republic. For HIV, the testing algorithm and clinical treatment guidelines have been revised to be in accordance with WHO guidelines and services have been brought closer to the population in PHC facilities. Moreover, rapid tests have been implemented in hospitals. For viral hepatitis B and C, testing is implemented in hospitals for health care workers (HCWs), pregnant women, patients (before surgery), and people living with HIV whereas testing services were paid out of pocket until 2023. Although rapid testing for viral hepatitis has been partially implemented, available evidence on coverage and uptake is scarce. As part of the national program implementation, awareness-raising of the importance of testing for the general population and key populations is planned, as well as an assessment of current practices. The political commitment is there along with the newly signed-off strategy with targets for improving testing. However, although a strategy and guidelines exist, the offer and uptake of HIV and viral hepatitis testing remain insufficient to reach the elimination targets and reduce the burden of disease in the country. While the opportunities are there, also for integrated HIV and viral hepatitis testing [13], more knowledge and data on barriers and facilitators are needed to foster and improve the implementation of testing.

Barriers are often described on 3 levels; system level, provider level, and patient level [14-16], but can also be analyzed using various frameworks, including the Capability, Opportunity, and Motivation model [17,18]. Our study will focus on the barriers and facilitators perceived by HCWs (provider level) to offer HIV and viral hepatitis testing in health care settings. This will be the first systematically collected overview and analysis of the most important barriers and facilitators to testing from the perspective of primary HCWs in the Kyrgyz Republic.

The overall aim of the study was to identify and describe the most important barriers and facilitators to HIV and viral hepatitis B, C, and D testing in primary health care settings in the Kyrgyz Republic.

This study aimed to answer the following research question following: What are the main barriers and facilitators for HIV and viral hepatitis testing in primary health care settings from the perspective of HCWs in the Kyrgyz Republic?

## Methods

### Study Team and Cooperation

The study was developed and carried out in collaboration with national public health authorities including the National Institute of Public Health of the Ministry of Health (MoH), the Republican Center for Viral Hepatitis, and HIV or AIDS Control at the MoH of the Kyrgyz Republic and professional networks of national study coordinators.

The local study partners were involved in the initial discussions and provided input to the protocol, study documents, and data collection tools. The local partners played a key role in study site selection. Local contextual knowledge and experience were crucial in this important first step of the study.

### Study Population

The target study population consisted of a broad range of HCWs including, but not limited to, physicians, nurses, and allied HCWs involved in the provision of HIV and viral hepatitis testing in primary health care settings.

### Study Design

**Figure 1:**
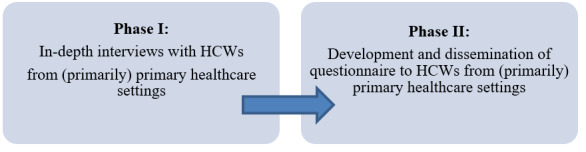
The 2 data collection phases of the study. HCW: health care worker.

A cross-sectional, mixed methods study was conducted to explore the views of HCWs working in primary health care settings in the Kyrgyz Republic on barriers and facilitators to HIV and viral hepatitis testing. We used multiple data collection methods and triangulation which included an overview of the current testing landscape, data collected through in-depth interviews with HCWs, and survey data collected through a questionnaire distributed among HCWs. The study was divided into 2 main phases (Figure 1).

Phase I included qualitative data collection and analysis by exploring the main barriers and facilitators perceived by HCWs in their testing roles. By conducting in-depth, semistructured interviews with a selected group of HCWs, we aimed to gather detailed information and their perspectives on the key barriers and facilitators to HIV and viral hepatitis testing in PHC settings. The method was chosen because it is particularly effective in exploring possibly sensitive topics such as HIV and viral hepatitis infection and service delivery practices, where personal experiences, attitudes, knowledge, and social contexts are crucial. The details obtained can provide insights into personal opinions, attitudes, behaviors, and motivations, information that is often not accessible through quantitative methods. In addition, in-depth interviews allowed us to maintain a degree of flexibility while probing more deeply into important areas that emerge during the interview [19-21]. The strength of phase I lies in its ability to uncover deeper, potentially overlooked insights by engaging directly with HCWs. This enabled a more detailed understanding of the complex dynamics of factors that influence testing practices [19-21].

Following phase I, the study moved to phase II, where a questionnaire was developed and distributed among HCWs in PHC settings. This questionnaire was designed based on the data from phase I and thereby collected more information from a wider range of HCWs. In addition to expanding the geographic coverage, it enabled an increase in the multitude and diversity of perspectives collected. The goal of this phase was to supplement and quantify the findings from phase I and to improve our understanding of the facilitators and barriers to testing among a broader group of HCWs.

### Study Sites

**Figure 2:**
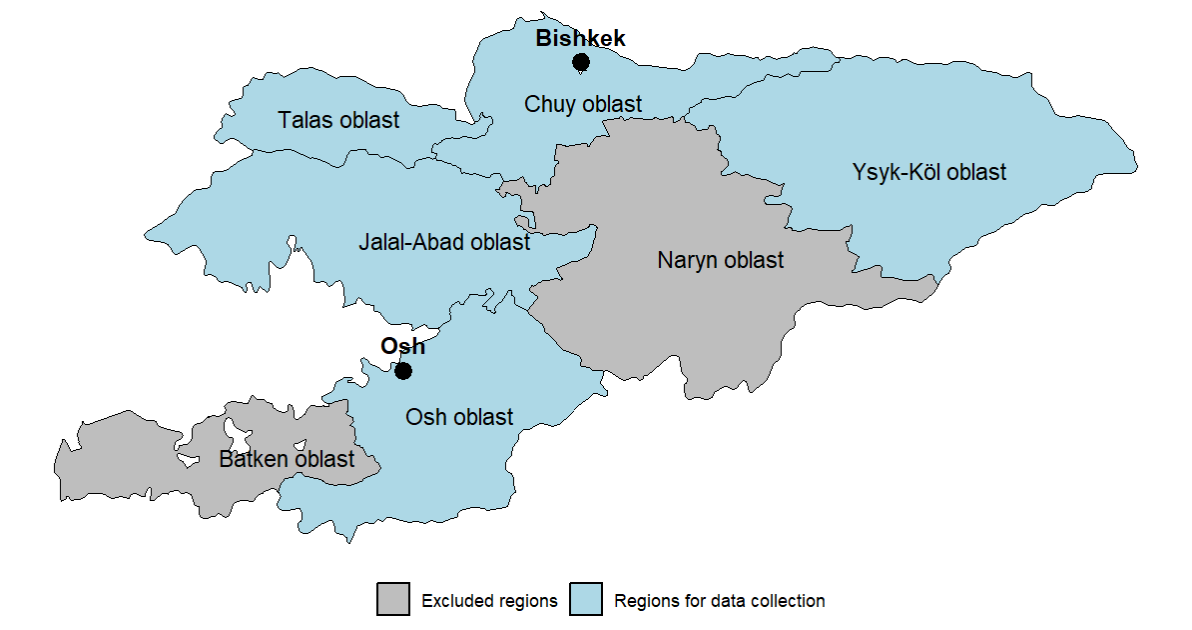
Overview of the 7 oblasts in the Kyrgyz Republic for data collection (phase 1 marked with blue).

The study sites from which HCWs were recruited included PHC and a few large specialized medical centers across the Kyrgyz Republic. There are in total 9 administrative regions (7 oblasts [Batken, Chui, Issyk-Kul, Jalal-Abad, Naryn, Osh oblast, and Talas] and 2 independent cities [Bishkek City and Osh City]) in the Kyrgyz Republic, with 44 districts (Figure 2).

For phase I, up to 15 sites were contacted that are located in different geographic areas, as well as sites located in both urban and rural settings from these 7 regions: Bishkek City, Chui, Issyk-Kul, Jalal-Abad, Osh City, Osh oblast, and Talas. The selection of the 7 regions is described in detail under the Sampling and Recruitment section.

For phase II, HCWs from all primary health care services in all of the 7 oblasts (Figure 2) were invited to take part in the survey. The selection of participants is explained in detail in the *Sampling and Recruitment* section.

### Sample Size

#### Phase I: In-Depth Interviews

The target sample size in phase I was up to 20 respondents. This size was considered a standard sample for qualitative studies of a medium size [19,22]. However, the final number of participants in this study was defined by the data saturation moment, which is the point where no new relevant insights on the research topic are received from our participants [19,22,23]. Data saturation was determined during the team meetings of the research group and data collectors. This approach ensured that the research process remained both efficient and focused by adjusting the scope of the study to match the quality of information collected, up to the point where further interviews would not add to the understanding of the research question [19,23].

#### Phase II: Questionnaire

By using a 95% confidence level and a 5% margin error, the sample should have been 385. Accounting for potential nonresponse, our target sample size in phase II was 400 participants.

### Sampling and Recruitment

#### Phase I: In-Depth Interviews

We recruited participants through the local study partners (see the *Study Team and Cooperation* section).

We used a multistage approach to select primary health care facilities in the country. Together with the National Institute of Public Health of the MoH, we went through their data including a complete list of primary health care facilities and their HIV and viral hepatitis testing volumes for 2023. This allowed us to conduct a geographical assessment using 3 key parameters: facility location, annual testing volume, and local population density. In order to identify facilities with the greatest need for assessment, we established a testing ratio by comparing each facility’s annual number of tests to the population density of its catchment area.

To supplement our facility selection process, we conducted confidential consultations with local civil society organizations that work closely with key populations affected by HIV and viral hepatitis. This allowed us to get information on reported experiences of stigma and discrimination in specific health facilities, which helped to contextualize potential barriers to testing services. Due to the sensitive sociopolitical environment and evolving regulatory framework affecting civil society organizations in the Kyrgyz Republic, we do not report identifying details about these consulting organizations to protect their operational security [24].

We sent the proposed list of facilities to the MoH for review and coordination. The national coordinators communicated with the MoH and selected facilities and provided them with detailed information about the study including contact information for the primary investigators, and confirming their capacity to participate within the proposed time frame. If a facility was unable to take part as a study site, another eligible facility from the same region was selected to ensure that all target regions were represented. This iterative selection process continued until we achieved our desired coverage of sites in the regions.

#### Phase II: Questionnaire

For the questionnaire, all health care services from the list of medical facilities provided by the partners in the Kyrgyz Republic were invited to take part in the internet-based survey and were asked to distribute the link to their HCWs. Probability-based sampling was not possible, as the exact number of HCWs in the different regions was unknown. However, due to the exhaustive content in the list of preselected health care services (N=77) covering all geographic areas, it was anticipated that the included study population would to a large extent be representative of HCWs in primary health care services in the regions. The electronic questionnaire was available through a link and was distributed via email and other preferred social and internet-based communication channels among the target population.

### Data Collection

Data collection consisted of a qualitative (phase I) and a quantitative (phase II) part, which is described in more detail below. Before the development of the semistructured interview guide and the following questionnaire, an overview of the testing landscape in the Kyrgyz Republic (in the form of a desk review) was conducted to understand by whom and where testing takes place. Questions and areas to which information was needed were developed by the Robert Koch Institute (RKI) study team and sent to the local partners to be completed. Answers and the final overview were provided and developed by local country partners to illustrate who should be tested according to national guidelines, which tests were available, as well as the current testing uptake and prevalence in the country (Multimedia Appendix 1). This context helped to inform the development of the interview guide and questionnaire.

#### Phase I: In-Depth Interviews

Data collection for phase I took place in June 2024. The semistructured interviews were conducted in both Russian and Kyrgyz depending on the preference of the participant (Multimedia Appendix 2). A total of 3 interviewers in total were involved in the data collection phase, but only 1 interviewer was present at each interview. The interviews lasted approximately 1 hour, face-to-face at a location chosen by the participant for their convenience and privacy. The interviews were audio recorded if permitted by the participants. A professional transcription service was contracted before data collection and transcribed the recordings using specialized software. To protect the anonymity of the participants, pseudonyms chosen by the participants were used in the transcripts. Finally, a certified translator translated the transcripts from Russian and Kyrgyz into English to ensure that high-quality data were accessible for analysis and reporting. The transcripts and their translation were proofread by the interviewer for data validation and verification.

#### Phase II: Questionnaire

The questionnaire was developed on the basis of the material and insights collected in phase I. A Likert scale (strongly agree, agree, disagree, and strongly disagree) was produced for the most frequently reported barriers and facilitators asking to what extent these were experienced by HCWs surveyed in phase II (Multimedia Appendix 3). The questionnaire also included a question on hepatitis B vaccination among HCWs (ever vaccinated, when and if partly or fully vaccinated). The questionnaire was available through the internet-based platform Voxco and accessible through a link and answers were saved directly through the survey-server Voxco.

### Analyses

#### Qualitative Analysis

Throughout the qualitative part, we followed the Consolidated Criteria for Reporting Qualitative Research [25]. The analysis of the collected data was conducted in English, taking advantage of the flexibility that problem-centered interviews offer, especially when exploring people’s experiences [19]. Given the focus of the study on individual experiences, multiple methods of analysis were considered. Our intention was not to generalize findings, but to look for patterns while remaining open to exploring the nuances, discrepancies, and potential conflicts inherent in the data [19].

Given our interest in identifying patterns of meaning in the data regarding barriers and facilitators to HIV and viral testing services, we used an inductive approach to reflexive thematic analysis [21,23]. This method is characterized by its adaptability and effectiveness in addressing different research questions and analyzing various types of data [21,23]. The inductive approach was chosen because it allowed us to develop codes and themes under the influence of the data, rather than being constrained by a predefined theoretical framework. In doing so, we ensured that all data collected, regardless of their initial perceived value, were included in the final analysis, providing a comprehensive understanding of the topic and allowing us to develop themes that the researchers were not aware of before embarking on data collection.

The data were analyzed using NVivo 14 software (released in 2023). A total of 2 researchers (IS and NL) conducted the analysis following Braun and Clarke’s [26] 6-step process for thematic analysis. In the first familiarization step, both researchers independently engaged in multiple readings of the interview transcripts to develop an understanding of the content and prepare the ground for analysis. Following familiarization, IS and NL proceeded with preliminary coding using the first 3 interview transcripts. To ensure analytical rigor, these transcripts were double-coded by both researchers. The coding process adopted an inductive approach. During this phase, both IS and NL identified and labeled segments of text relevant to understanding barriers and facilitators to HIV and viral hepatitis testing. The initial coding was done independently to ensure that both researchers identified a wide range of codes. Once the initial coding was completed, the 2 researchers discussed and refined the initial codes and merged overlapping concepts to develop a consolidated coding framework which was applied to the remaining transcripts, moving from an inductive to a more structured deductive approach. In the third step, the codes were organized into potential themes that involved grouping related codes to form overarching themes that capture the essence of the data. After identifying potential themes, in step 4 IS and NL shared evolving codes and themes with the core research team for discussion and refinement to ensure the reliability and validity of the analysis.

Once the final themes were defined, the 2 researchers defined and named subthemes to capture specific nuances of each theme, and then completed the final analytical step involving synthesizing the themes into a narrative story [27] which addressed the research question.

The background and previous experience of the researchers influenced the study from the development of ideas and data collection tools, to the analysis of data. Personal reflexivity shaped the analytical steps and is important to reflect on [27]. The first and second authors who carried out the 6 steps of the thematic analysis have different professional backgrounds and views and thereby a natural step of the analysis process was to use reflexive practices to align and discuss decisions during both developmental and analytical steps of the research.

The first author (IS) has a public health background specializing in infectious disease epidemiology and has experience with viral hepatitis and HIV. Her previous experience mainly with key populations might have influenced a particular focus on and interest for these groups, however knowing that the experience primarily comes from a European context.

The second author (NL) brings a dual perspective to this research as both a medical professional and a community member, with clinical experience in HIV medicine and a background in global health research with active use of the qualitative methodology. As a gay man from Eastern Europe with cultural ties to Central Asia, he has insider knowledge of the needs of Gay, bisexual, and other men who have sex with men communities in the region. This dual role as both health care provider and service user strengthened data collection and analysis.

To achieve trustworthiness in qualitative research, it is important to reflect on credibility which we in this study tried to achieve by extended involvement and discussion within the larger research team as well as triangulation with observations during the interviews. To achieve transferability, the methods will be meticulously described and explained for readers to be able to follow all steps. Dependability and confirmability will be ensured by thorough documentation of steps and through notes and confirmability through discussion with both project partners in the Kyrgyz Republic, but also with the wider project team.

#### Quantitative Analysis

The analyses from the questionnaire will be conducted in R 4.2.2 (with the packages “dplyr” and “epitools”). The analyses will be descriptive. We will derive frequencies for categorical variables, including the most frequently reported barriers and facilitators for HIV and viral hepatitis testing.

If the number of respondents and available data are sufficient to ensure statistical validity, stratified analyses will be conducted to explore differences in barriers and facilitators across subgroups using chi-square tests. As a start, the data will be stratified according to the following:

1. Geography: urban and rural region
2. Type of HCW: specialty and background
3. Age of participants
4. Sex: male and female

Other variables that may also be of interest include knowledge and training, experience with key populations, and availability of free tests as well as awareness of current testing guidelines.

#### Quality Assurance

The protocol was reviewed by the entire project team including the project partners in the Kyrgyz Republic. The semistructured interview guide developed for the interviews was checked and evaluated by the project team members and piloted with one HCW from the Kyrgyz Republic in order to also control for a possible translation bias. The interview guide was revised based on the feedback provided.

### Ethical Considerations

Ethics approval was obtained from the institutional review board of Scientific and Practical Health Care in Kyrgyzstan in Bishkek, Kyrgyz Republic (01-3/15; May 23, 2024). A study information sheet was provided to potential participants (in phases I and II) and written informed consent was collected before participation. Participants in phase I were also asked to consent to be quoted in publications and had the option to only consent to participation (and no quotes in publications). The informed consent form included information about study objectives, and data protection (procedure and rights), including how long and where data will be stored and who has access. The form also included information on the person responsible for the study and the person responsible for data protection. The participant was informed of their rights to withdraw participation at any time without negative consequences. All participants had to be older than 18 years of age. Privacy and confidentiality were ensured by a review of the acquisition and processing procedures provided by the RKI data protection officer. The in-depth interviews were recorded and transcripts were produced in the Kyrgyz Republic. Only anonymized written text was shared with RKI through the secure server Cryptshare. After the transfer of the interviews to a secure server, they were deleted from the recording devices. All personal data will be deleted after the end of the study.

The informed consent forms will be kept at RKI until the end of the study and it will not be possible to link the data to the personal information (ie, names of participants). All personal data will be deleted after the end of the study.

A list of participants in phase I was needed for the purpose of providing incentives for participation. This list will be deleted 6 years after the end of the study.

Participants in the in-depth interviews were reimbursed with a financial incentive for their time, transport, and participation of 2000 Soms (equivalent to around €22 [US $24.58]).

## Results

Data collection took place in June 2024 for phase I and in November 2024 for phase II. Data analyses and writing up of results will be done in early 2025 and results are expected to be published in spring 2025.

## Discussion

The new national program on the elimination of HIV and viral hepatitis (2023-2027) in the Kyrgyz Republic demonstrates a political will to improve the response to these 2 diseases. Important content of the program addresses improving testing and decentralizing care, moving testing from specialist to primary HCWs. While this task shift makes sense in theory, there may be various reasons why the implementation is challenging. It is anticipated there will be more barriers to HIV testing, in particular among key populations, due to lack of experience and training, but also stigma and discrimination against certain population groups. Moreover, this is expected to be more prevalent in rural areas. Viral hepatitis is expected to be less stigmatized as it is more common among the general population and barriers here might be more related to lack of time or competing priorities.

An important strength of this study is that the protocol and data collection instruments were reviewed by a larger study team as well as local partners with contextual knowledge. The involvement of already trusted local partners was important in order to ensure successful implementation and distribution, and approval from partners ensured support from the MoH, and also primary health care settings who took part. The testing landscape was a good foundation for all further methodological steps of the study. Moreover, the triangulation of contextual background information in the form of a testing landscape, as well as our mixed methods approach was valuable in creating both a deep, but also nuanced understanding of testing activities and important barriers and facilitators in the country.

Despite important strengths, our study will also be subject to challenges and limitations. There is a risk of selection bias in case some participants did not have the capacity or time to participate. It may also be that the HCWs will appreciate the opportunity to express their concerns or ideas on the topic. Careful instructions at the beginning of the interviews, securing anonymity and patience throughout the interview hopefully provided a safe space to express their views on barriers and facilitators.

For the questionnaire, a nonrandomized sampling of HCWs might have created bias as some groups may be underrepresented providing a skewed picture of barriers and facilitators for testing. Involving the MoH in recruitment might have ensured a large number of participants, but may also have affected the information provided by the participants, despite anonymity, and they may have been prone to report what they think they are expected to report. The involvement of local partners was a strength, but may also be a weakness as it can influence who takes part and what they report, as the study team at RKI is not independent of local constructs in the country. A lot of testing in the country takes place in specialized settings, such as hospitals, clinics for sexually transmitted infections, and blood centers. Moreover, community testing as well as self-testing has been rolled out in the country. However, due to time, budget, and staff limitations it was necessary to narrow down the scope of the study.

With this study, we will be able to report on important barriers and facilitators, both detailed and in-depth through interviews, but also from a large study population (HCWs) through the questionnaire. The study will hopefully highlight reasons for gaps in testing and be able to provide recommendations for how to improve testing offered in primary health care in the Kyrgyz Republic.

## Appendix

Multimedia Appendix 1 Testing landscape.

Multimedia Appendix 2 Semistructured interview guide.

Multimedia Appendix 3 Online questionnaire.

## Abbreviations

HCW: health care worker
MoH: Ministry of Health
PHC: primary health care
RKI: Robert Koch Institute
WHO: World Health Organization

## Data Availability

The datasets generated during and/or analyzed during this study are not publicly available due to data protection, particularly for the qualitative data (phase I), but are available from the corresponding author upon reasonable request.

## References

[1] (2022). Hepatitis B in the WHO European Region - fact sheet July. World Health Organization.

[2] (2022). Hepatitis C in the WHO European Region - fact sheet July. World Health Organization.

[3] World Health Organization (2024). Global Hepatitis Report 2024: Action for Access in Low- and Middle-Income Countries.

[4] Davlidova S, Haley-Johnson Z, Nyhan K, Farooq A, Vermund SH, Ali S (2021). Prevalence of HIV, HCV and HBV in Central Asia and the Caucasus: a systematic review. Int J Infect Dis.

[5] (2021). Global progress report on HIV, viral hepatitis and sexually transmitted infections. World Health Organization.

[6] World Health Organization (2022). Consolidated Guidelines on HIV, Viral Hepatitis and STI Prevention, Diagnosis, Treatment and Care for Key Population.

[7] (2024). HIV/AIDS. World Health Organization.

[8] (2024). HIV/AIDS surveillance in Europe 2024 – 2023 data. WHO Regional Office for Europe ECfDPaC.

[9] World Health Organization (2023). Regional Action Plans for Ending AIDS and the Epidemics of Viral Hepatitis and Sexually Transmitted Infections 2022–2030.

[10] (2022). HIV and viral hepatitis programme review in Kyrgyzstan. World Health Organization.

[11] Bogers S, Boyd A, Schim van der Loeff M, Geerlings S, Davidovich U, HIV Transmission Elimination AMsterdam (and H-TEAM) Consortium (2024). Opportunities for improved indicator-based HIV testing in the hospital setting: a structural equation model analysis. AIDS Care.

[12] Sullivan AK, Raben D, Reekie J, Rayment M, Mocroft A, Esser S, Leon A, Begovac J, Brinkman K, Zangerle R, Grzeszczuk A, Vassilenko A, Hadziosmanovic V, Krasnov M, Sönnerborg A, Clumeck N, Gatell J, Gazzard B, Monforte AD, Rockstroh J, Lundgren JD (2013). Feasibility and effectiveness of indicator condition-guided testing for HIV: results from HIDES I (HIV indicator diseases across Europe study). PLoS One.

[13] Dara MS, Ehsani S, Mozalevskis A, Vovc E, Simões D, Avellon Calvo A, Casabona I Barbarà J, Chokoshvili O, Felker I, Hoffner S, Kalmambetova G, Noroc E, Shubladze N, Skrahina A, Tahirli R, Tsertsvadze T, Drobniewski F (2020). Tuberculosis, HIV, and viral hepatitis diagnostics in eastern Europe and central Asia: high time for integrated and people-centred services. Lancet Infect Dis.

[14] Deblonde J, De Koker P, Hamers FF, Fontaine J, Luchters S, Temmerman M (2010). Barriers to HIV testing in Europe: a systematic review. Eur J Public Health.

[15] Mason LMK, Veldhuijzen IK, Duffell E, van Ahee A, Bunge EM, Amato-Gauci AJ, Tavoschi L (2019). Hepatitis B and C testing strategies in healthcare and community settings in the EU/EEA: a systematic review. J Viral Hepat.

[16] Bagchi AD, Davis T (2020). Clinician barriers and facilitators to routine HIV testing: a systematic review of the literature. J Int Assoc Provid AIDS Care.

[17] Khanna R, Gobin M (2024). Application of the COM-B model to facilitators and barriers to HIV and STI testing among people from Black African and Black Caribbean communities in the UK: a scoping review. Sex Transm Infect.

[18] Michie S, van Stralen MM, West R (2011). The behaviour change wheel: a new method for characterising and designing behaviour change interventions. Implement Sci.

[19] Witzel A, Reiter H (2012). The Problem-Centred Interview.

[20] Braun V, Clarke V (2006). Using thematic analysis in psychology. Qual Res Psychol.

[21] Braun V, Clarke V (2019). Reflecting on reflexive thematic analysis. Qual Res Sport Exerc Health.

[22] Bryman A (2007). Barriers to integrating quantitative and qualitative research. J Mix Methods Res.

[23] Terry G, Hayfield N, Clarke V, Braun V (2017). Thematic analysis. The SAGE Handbook of Qualitative Research in Psychology.

[24] (2023). Statement: Kyrgyzstan targets LGBTI communities in a new law 2023. ILGA-Europe.

[25] Tong A, Sainsbury P, Craig J (2007). Consolidated criteria for reporting qualitative research (COREQ): a 32-item checklist for interviews and focus groups. Int J Qual Health Care.

[26] Braun V, Clarke V (2006). Using thematic analysis in psychology. Qual Res Psychol.

[27] Braun V, Clarke V (2023). Toward good practice in thematic analysis: Avoiding common problems and be(com)ing a researcher. Int J Transgend Health.

