# Supplementary material for: Barriers and Facilitators to HIV and Viral Hepatitis Testing in Primary Health Care Settings in the Kyrgyz Republic (BarTest): Protocol for a Mixed Methods Study
Source: JMIR Res Protoc. 2025 May 28;14:e62929. doi: 10.2196/62929 (PMC12159551; doi:10.2196/62929)
Supplement: Multimedia Appendix 1 [file resprot_v14i1e62929_app1.docx]

Welcome and thank you for participating in this interview!

Your insights and experiences are incredibly valuable to our research. This interview is part of a study being conducted by the National Public Health Institute (Kyrgyzstan) and Robert Koch Institute (the National Public Health Institute in Germany) to identify and describe the most important barriers and facilitators to HIV and viral hepatitis B, C and D testing in primary health care settings in Kyrgyzstan.

The purpose of this interview is to collect information about your personal experiences, perspectives and thoughts regarding HIV and viral hepatitis B, C and D testing. There are no right or wrong answers, and your perspective will help us better understand the challenges and opportunities faced by healthcare professionals like you, and will contribute to the development of more effective and responsive public health interventions and policies.

My name is [your name] and I am going to conduct this interview with you today. Our interview will take approximately 1 hour. You may choose to skip any questions you are uncomfortable answering, and you may withdraw from the interview at any time without consequence. With your permission, we would like to record the interview to ensure that we capture your answers accurately. The recordings will be kept securely and used for research purposes only.

**Section 1: Opening questions**

- Could you please describe your daily work with providing healthcare services, specifically HIV and viral hepatitis testing?
- Do you think that HIV and viral hepatitis are large challenges in your country?

**Section 2: Testing Practices (by HCW in their setting)**

- Can you walk me through the steps for HIV and viral hepatitis testing in your facility?
- How is your experience with offering HIV/Hep test to patients?
  - To which patients/in what situations would you offer an HIV/hep test?
  - Do you feel comfortable talking to patients about risk factors for HIV/hepatitis which warrants testing (reference to strategy)?
  - (*If not much experience/not offering tests*) What are the main reasons for not offering a test?

*Probes*:

- - Only tests if patient asks to be tested
  - Lack of time?
  - Shortage of staff?
  - Lack of knowledge?
  - Doesn’t want to deal with such patients?
  - Any other logistical or infrastructural challenges?
  - Do not see it as their responsibility? (primary healthcare settings)
  - Other diseases “more” important?
- Are rapid tests available in your healthcare setting?
  - How would you describe the role of HIV/hep rapid tests in your setting?
- How is the follow-up with patients conducted post-testing, especially in cases of positive results (linkage to care/treatment pathway)?
  - What is your personal experience? *(See if they feel comfortable to talk about positive results with patients) (probe: ask something about if lack to access to treatment/not knowing what to do with a positive patient, does that sometimes prevent testing)*

**Section 3: Information on testing for the HCW**

- How did your (medical) studies prepare you for offering HIV/hep tests?

*Probes*:

- - - - Need/role for additional training?
      - How often?
      - By whom?
- How do you keep yourself updated in terms of who to offer an HIV/hep test?

*Probes:*

- - Decrees, staff updates, written information from head of the clinic

**Section 4: Barriers to Testing**

- In your experience, what are the most important reasons why healthcare workers do not offer testing for HIV and viral hepatitis to patients?
- Which factors influence attitudes towards testing among your colleagues?

*Probes:*

- - language
  - gender identity of doctors vs. Gender identity of patients
  - sexual identity of patients
  - lack of trust to doctors or low awareness
- Are there any key populations that have more difficulties accessing testing? Can you elaborate on why that might be the case?

*Probes:*

- - People who use drugs?
  - Gay, bisexual and other MSM?
  - Trans* people?
  - Sex workers?
  - Working migrants or family members of working migrants
- In your opinion, how does the legal and policy environment towards key populations impact HIV and viral hepatitis testing?

*Probes:*

- - Free testing
  - LGBT propaganda law
  - Substance use is partially criminalized
- Do stigma and discrimination play a role in deterring individuals from getting tested? How is this addressed in your practice?

**Section 5: Facilitators for Testing**

- Have there been any initiatives/ideas improved testing rates for HIV and viral hepatitis in your setting? Please elaborate.

*Probes:*

- - Free testing
  - Link from NGOs (training, information to patients from other organizations..)
  - Support from government/international organizations
- What do you think could improve testing rates for HIV and viral hepatitis in your settings? *Probes:*
  - technological advancements?
  - Awareness raising among population? Among doctors?

**Section 6: Closing questions**

- Are there any specific aspects around HIV/Hep testing that we did not cover that you would like to add?
